# MLST reveals a clonal population structure for *Cryptococcus neoformans* molecular type VNI isolates from clinical sources in Amazonas, Northern-Brazil

**DOI:** 10.1371/journal.pone.0197841

**Published:** 2018-06-08

**Authors:** Diego Fernando Silva Rocha, Katia Santana Cruz, Carla Silvana da Silva Santos, Lizandra Stephanny Fernandes Menescal, João Ricardo da Silva Neto, Silviane Bezerra Pinheiro, Lucyane Mendes Silva, Luciana Trilles, João Vicente Braga de Souza

**Affiliations:** 1 Medical Mycology Laboratory, Tropical Medicine Foundation Dr. Heitor Vieira Dourado, Manaus, Amazonas, Brazil; 2 Mycology Laboratory, Coordination of Society, Environment and Health of National Research Institute of Amazonia, Manaus, Amazonas, Brazil; 3 National Institute of Infectology Evandro Chagas, Oswaldo Cruz Foundation, Rio de Janeiro city, Rio de Janeiro, Brazil; University of Birmingham, UNITED KINGDOM

## Abstract

Cryptococcosis is considered endemic in Amazonas state, occurring more frequently in individuals with AIDS, who are predominantly infected by *Cryptococcus neoformans* molecular type VNI. Infections by *Cryptococcus gattii* VGII predominate in immunocompetent hosts from the American continent and are associated with outbreaks in North America, particularly the subtypes VGIIa and VGIIb, which are also present in the Brazilian Amazon region. Despite few environmental studies, several aspects of the molecular epidemiology of this disease in Amazonas remain unclear, including the limited use of multilocus sequence typing (MLST) to evaluate the genetic population structure of clinical isolates, mainly *C*. *neoformans*. Therefore, we used MLST to identify the sequence types of 38 clinical isolates of *C*. *neoformans* VNI and *C*. *gattii* VGII and used phylogenetic analysis to evaluate their genetic relationship to global isolates. Records of 30 patients were analyzed to describe the current scenario of cryptococcosis in the region and their associations with the different subtypes. Broth microdilution was also performed to determine the susceptibility profile to the antifungals amphotericin B, fluconazole and itraconazole. MLST identified that patients with HIV (n = 26) were exclusively affected by VNI strains with ST93, and among the VGII strains (n *=* 4), three STs (ST5, ST172 and the new ST445) were identified. An in-hospital lethality of 54% was observed in the HIV group, and there were no significant differences in the clinical aspects of the disease between the HIV and non-HIV groups of patients. In addition, all isolates were susceptible to the antifungals tested. Therefore, in Amazonas state, VNI isolates are a genetically monotypic group, with ST93 being highly important in HIV individuals.

## Introduction

Cryptococcosis is a systemic, cosmopolitan and primary opportunistic infection associated with human immunodeficiency virus (HIV), representing an important public health problem [1–4]. Both the pathogens *Cryptococcus neoformans* and *Cryptococcus gattii* constitute species complexes widely distributed in the environment, mainly associated with bird feces and wood debris [5–9]. The disease is acquired by inhalation of infectious propagules from the environment (desiccated yeasts cells or basidiospores) and its most frequent clinical manifestation is meningoencephalitis [10–13].

*C*. *neoformans* is the most frequently isolated species in individuals with HIV, causing an estimated annual occurrence of 223,100 cases of cryptococcal meningitis worldwide, resulting in approximately 181,100 deaths [14]. Cryptococcosis is a non-reportable disease in Brazil and correct estimates on the epidemiology of this infection are scarce. However, data from the literature and the Brazilian Ministry of Health revealed that approximately 7,000 cases of cryptococcal meningoencephalitis are diagnosed annually, mainly in the southeast region, of which 90% occur in patients with AIDS, whose death rate associated with cryptococcosis is approximately 35–40% [15,16].

By comparison, *C*. *gattii* is more commonly found in Australia, the Pacific Northwest of North America and Northern parts of South America, and it is considered a primary infection agent, although there is evidence that intrinsic deficiencies in specific defense mechanisms may predispose certain individuals to *C*. *gattii* [17,18].

The genotypic variability of cryptococcosis agents has been investigated worldwide to detect polymorphisms in DNA using several PCR techniques that initially allowed the identification of the following main molecular types: VNI/AFLP1, VNII/AFLP1A, VNB/AFLP1B, VNIII/AFLP3, and VNIV/AFLP2 for *C*. *neoformans;* and VGI/AFLP4, VGII/AFLP6, VGIII/AFLP5, and VGIV/AFLP7 for *C*. *gattii* [19,20]. To globally standardize the genotyping of the *C*. *neoformans/C*. *gattii* species complex, a MLST scheme was established by the International Society for Human and Animal Mycology (ISHAM) working group “Genotyping *C*. *neoformans* and *C*. *gattii*” based on variable regions within the capsular associated protein gene (*CAP59*), glyceraldehyde-3-phosphate dehydrogenase gene (*GPD1*), laccase (*LAC1*), phospholipase (*PLB1*), Cu, Zn superoxide dismutase (*SOD1*), orotidine monophosphate pyrophosphorylase (*URA5*) gene and the intergenic spacer region (IGS1), for their high discriminatory power and good reproducibility between different laboratories. The subtypes are defined via an online database (http://mlst.mycologylab.org) and are called sequence types (STs) [21].

Sequence types of *C*. *gattii* VGII were previously identified in the Amazonas, most of them from the environment [22,23]; however, there is no published data about the molecular population structure of *C*. *neoformans* VNI from the region. Clinical isolates with resistance to fluconazole were previously detected in the Amazonas state; therefore, surveillance studies of subtypes and antifungal susceptibility are crucially important [24]. Thus, the current study used MLST to identify the sequence types of 38 clinical isolates of *C*. *neoformans* VNI and *C*. *gattii* VGII from Amazonas state and to evaluate their genetic relationship with global isolates. Additionally, we determined the susceptibility profile to the antifungal agents amphotericin B (AMB), fluconazole (FLZ) and itraconazole (ITZ) and described the clinical and epidemiological characteristics of patients to evaluate the current scenario of cryptococcosis in the region.

## Materials and methods

### Clinical isolates

A total of 38 clinical isolates of *Cryptococcus*, including 34 *C*. *neoformans* and four *C*. *gattii*, were recovered from cerebrospinal fluid (CSF) samples (n = 30) and blood cultures (n = 8) obtained from 30 patients hospitalized between February 2014 to May 2016 at the Tropical Medicine Foundation Dr. Heitor Vieira Dourado [Fundação de Medicina Tropical Dr. Heitor Vieira Dourado (FMT-HVD)] in Manaus, Amazonas state (AM), Brazil. All isolates were maintained in Sabouraud dextrose agar tubes and stored at 4°C at the Medical Mycology Laboratory at FMT-HVD. The strains were purified twice on niger seed plates, and then only one isolated colony was randomly selected for further analysis. Eight serial isolates were recovered from the CSF samples of two patients, and isolates from CSF and blood were recovered from two patients (S1 Table).

### Collection of epidemiological and laboratory data

Clinical, epidemiological and laboratory records of all patients were accessed from the online database of FMT-HVD. The data collected for analysis included age, gender, geographic location, initial symptoms and developed sequelae, HIV infection status, CD4^+^ T cell count (at the time of diagnosis), clinical outcome (death or survival), need for surgical intervention and hospitalization in the intensive care unit (ICU) due to complications, clinical forms, time and number of hospitalizations and the amount of positive cultures recovered in the initial diagnosis and during treatment. This study was approved by the FMT-HVD Human Research Ethical Committee (CAAE 53952416.1.0000.0005). Patients enrolled in the study provided their written informed consent, and data were analyzed anonymously.

### Molecular typing by *URA5*-RFLP

DNA extraction was performed using the phenol:chloroform:isoamyl-alcohol method [25]. The major molecular types were first determined by *URA5*-RFLP analysis with Sau96I and HhaI (Thermo Scientific, Waltham, USA) enzymes as described by Meyer et al. (2003) [20]. The genotypes were assigned by comparison with the respective reference strains: WM 148 (serotype A, VNI), WM 626 (serotype A, VNII), WM 628 (serotype AD, VNIII), WM 629 (serotype D, VNIV), WM 179 (serotype B, VGI), WM 178 (serotype B, VGII), WM 161 (serotype B, VGIII) and WM 779 (serotype C, VGIV).

### MLST and phylogenetic analysis

MLST analysis was performed by the individual amplification of the six housekeeping genes *CAP59*, *GPD1*, *LAC1*, *PLB1*, *SOD1*, *and URA5* along with the IGS1 region according to the conditions published previously by the ISHAM [21]. The PCR products were purified with a modified method using polyethylene-glycol/NaCl [26] and were bidirectionally sequenced on an ABI3130 DNA Analyzer with BigDye Terminators v3.1 (Applied Biosystems, Foster City, California, USA) at the Laboratory of Functional Genomic and Bioinformatics (Fiocruz, Rio de Janeiro, Brazil). The sequences were manually edited using the software Sequencher 5.3 (Gene Codes Corporation, Ann Arbor, MI, USA), and the contigs were aligned using the Muscle algorithm linked to the program MEGA v6.06 [27]. All sequences were analyzed in the MLST for *C*. *neoformans* and *C*. *gattii* species complex database (http://mlst.mycologylab.org) to determine the allele number and respective ST. The sequences were deposited in GenBank and the accession numbers can be found in the S1 Table.

Using the software MEGA v6.06, the concatenated DNA sequences of seven MLST loci from clinical isolates were aligned by Muscle along with the sequences of VNI (*n* = 173) and VGII (*n* = 167) STs available in the Fungal MLST Database. To verify the genetic and evolutionary relationship among these STs, a phylogenetic tree was constructed based on the neighbor-joining (NJ) model with a bootstrap analysis using 1,000 replicates. The evolutionary distances were computed using the p-distance and all gaps were eliminated. Due to the large number of STs present in the database, a second phylogenetic tree was constructed using the same methods described above with only subsets of genetically closely related STs, retaining mainly the STs previously identified in Amazonas to analyze their genetic association [22,23].

### Antifungal susceptibility test

The antifungal susceptibility test was performed using the microdilution method in RPMI broth according to the M27-A3 guideline of the Clinical and Laboratory Standards Institute (CLSI) [28]. The microdilution of drugs tested was performed in duplicate and in the following ranges: 0.125–64 μg/ml for FLZ (Iberoquímica Magistral, Jundiaí, Brazil) and 0.03–16 μg/ml for AMB (Sigma Aldrich, Saint Louis, USA) and ITZ (Sigma Aldrich, Saint Louis, USA).

*Cryptococcus* isolates were subcultured onto Sabouraud dextrose agar and incubated for 48 h at 35°C. The yeast colonies were transferred to 5 ml of sterile saline solution (0.85%) and adjusted to a density equivalent to 0.5 McFarland standard scale. The inoculum was adjusted to 2.5 × 10^3^ cells in 10 ml of RPMI medium (Sigma Aldrich, Saint Louis, USA) by counting in a Neubauer chamber. The 96-well microplates were incubated at 35°C for 72 h. The MIC of amphotericin B was determined as the lowest concentration that completely inhibited fungal growth (100%), and for the azoles, the lowest concentration that generated partial reduction (50%) compared with the growth-control wells. The interpretation of MIC values was based on the following genotype-specific epidemiological cut-off values (ECVs): AMB (0.5 μg/ml), FLZ (8 μg/ml) and ITZ (0.25 μg/ml) for VNI; and AMB (1 μg/ml), FLZ (32 μg/ml) and ITZ (0.5 μg/ml) for VGII strains [29,30].

### Statistical analysis

Statistical data were analyzed with R Software version 3.3.1 (https://www.r-project.org) and described using the relative frequency, mean and standard deviation. The variables were compared between groups defined according to HIV infection status and the corresponding infecting species.

## Results

### Clinical and epidemiological data

**Fig 1:**
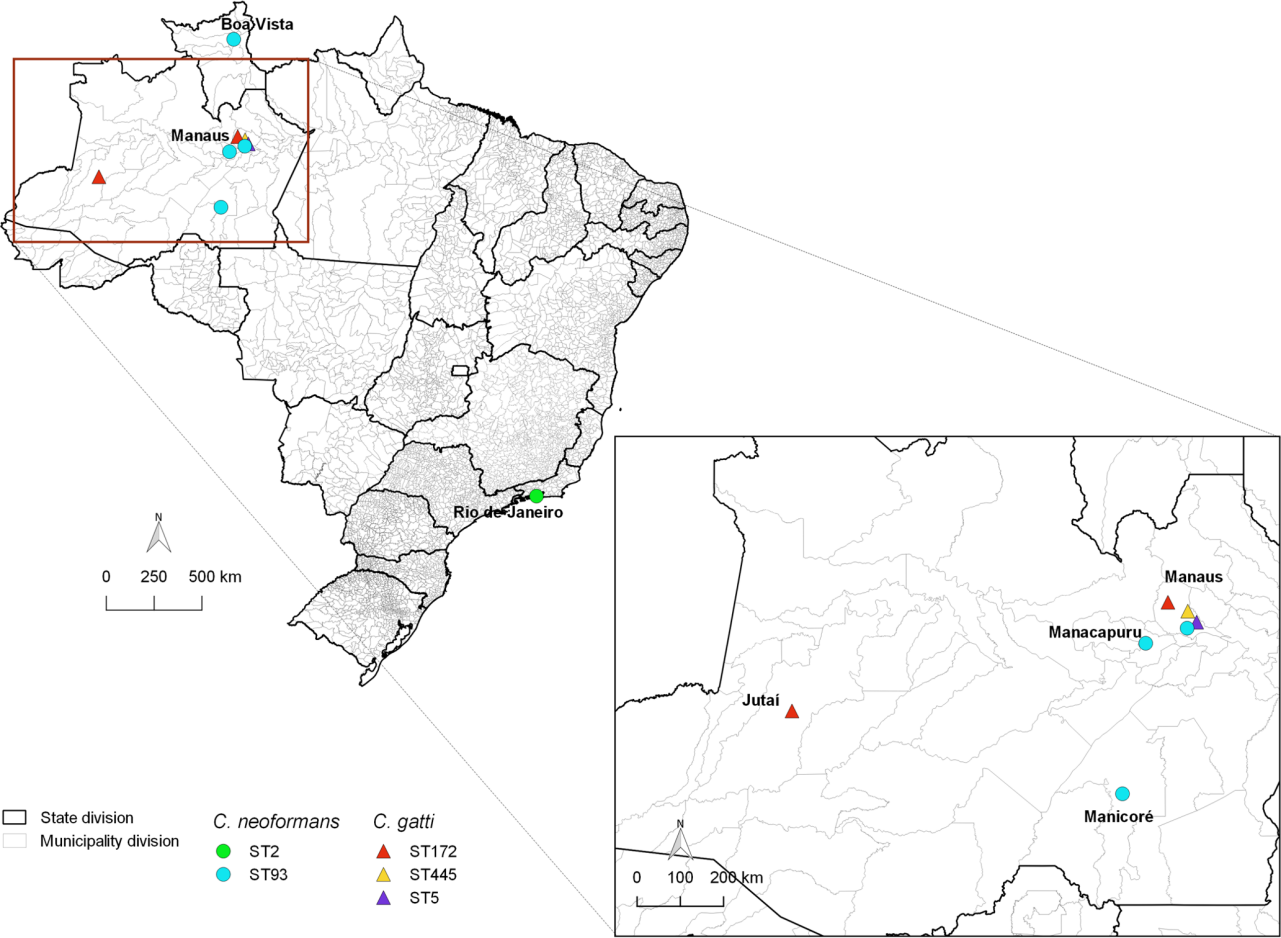
Map of Brazil showing the origin of the 30 patients studied and the corresponding infecting molecular types. *C*. *neoformans* VNI and *C*. *gattii* VGII were indicated by circle and triangle shapes and sequence types by different colors. QGIS v.2.16.1 software was used to construct the map.

Clinical, epidemiological and laboratory data were obtained for the 30 patients investigated. Most were from Manaus (25; 83%) while some were from other municipalities, such as Manacapuru (1; 3%) in the metropolitan region, Manicoré (1; 3%) and Jutaí city (1; 3%) located, respectively, in the south and southwest of Amazonas. Two non-autochthonous cases were also diagnosed at FMT-HVD, one from Boa Vista (Roraima State–North of Brazil) and the other from Rio de Janeiro (Southeast of Brazil) (Fig 1).

The majority of patients were male (19; 73%), and the mean age was 39.8 ± 12.2 years with a range of 19–68 years. HIV infection was reported for 26 patients (87%), of which 17 (74%) presented CD4^+^ T-cell counts below 50 mm^3^/ml, and all were affected only by *C*. *neoformans* VNI. Neurocryptococcosis was the most frequent clinical presentation (29; 97%), and of these cases, 10 (34%) were also associated with blood infection; only one HIV patient had fungemia alone. The most common initial signs and symptoms were headache (28; 93%), fever (21; 70%), weight loss (17; 57%), disorientation (14; 47%) and visual impairment (11; 37%). Four (13%) patients also used CSF derivation systems to overcome intracranial hypertension. Neurological sequelae, such as decreased visual acuity (10; 33%), hearing deficit (4; 13%), motor deficit (3; 10%), hydrocephalus (3; 10%) and hyposmia (2; 7%), were the most frequent. Moreover, in-hospital death was observed for half of the patients (15; 50%), mainly those with HIV (14; 54%), during the first 100 days after admission (Table 1). The only four apparently immunocompetent patients were infected with *C*. *gattii* VGII.

**Table 1:** Comparison of clinical, epidemiological and laboratory features of patients with cryptococcosis in Amazonas according to the HIV infection status.

Variables	HIV positive *C*. *neoformans* VNI *N =* 26 (%)	Non-HIV *C*. *gattii* VGII *N =* 4 (%)
**Demographics**		
Male sex	19 (73)	1 (25)
Age in years (Mean ± SD)	39.2 ± 12.3	44.5 ± 12.1
Age (Range)	19–68	30–55
**Clinical presentation at baseline**		
Headache	24 (92)	4 (100)
Nausea/Vomiting	18 (73)	4 (100)
Fever	18 (69)	3 (75)
Weight loss	14 (54)	3 (75)
Disorientation	12 (46)	2 (50)
Visual deficit	7 (27)	4 (100)
Cough	7 (27)	2 (50)
Seizure	6 (23)	1 (25)
Dizziness	6 (23)	1 (25)
Dyspnea	4 (15)	1 (25)
Photophobia	4 (15)	1 (25)
Meningeal signals	3 (11.5)	1 (25)
Papilledema	1 (4)	1 (25)
**CD4**^**+**^ **T cells/mm**^**3**^	23 (88.5)	-
> 50 cells/mm^3^	6 (26)	-
< 50 cells/mm^3^	17 (74)	-
**Clinical forms**		
Neurocryptococcosis	15 (58)	4 (100)
Neurocryptococcosis and fungemia	10 (38)	-
Fungemia	1 (4)	-
**Positive cultures** (Mean ± SD)	2 (1–3.8)	1.5 (1–2.2)
**Hospitalizations** (Mean ± SD)	1 (1–3)	1 (1–1.2)
**Hospitalization Time** Days (Mean)	57 (36.2–84)	57(48.8–67.5)
**Need of CSF shunt**	3 (11.5)	1 (25)
**Outcome**		
Death	14 (54)	1 (25)
Hospital discharge	12 (46)	3 (75)
**Admission to death** (time in days)		
< 100	7 (50)	1 (100)
101–200	1 (7)	-
>200	6 (43)	-
**Sequels**		
Decreased visual acuity	10 (38)	1 (25)
Decreased hearing acuity	4 (15)	-
Motor deficit	3 (11.5)	-
Hydrocephalus	3 (11.5)	-
Hyposmia	2 (8)	-

SD: standard deviation.

### MLST and phylogenetic analysis

**Fig 2:**
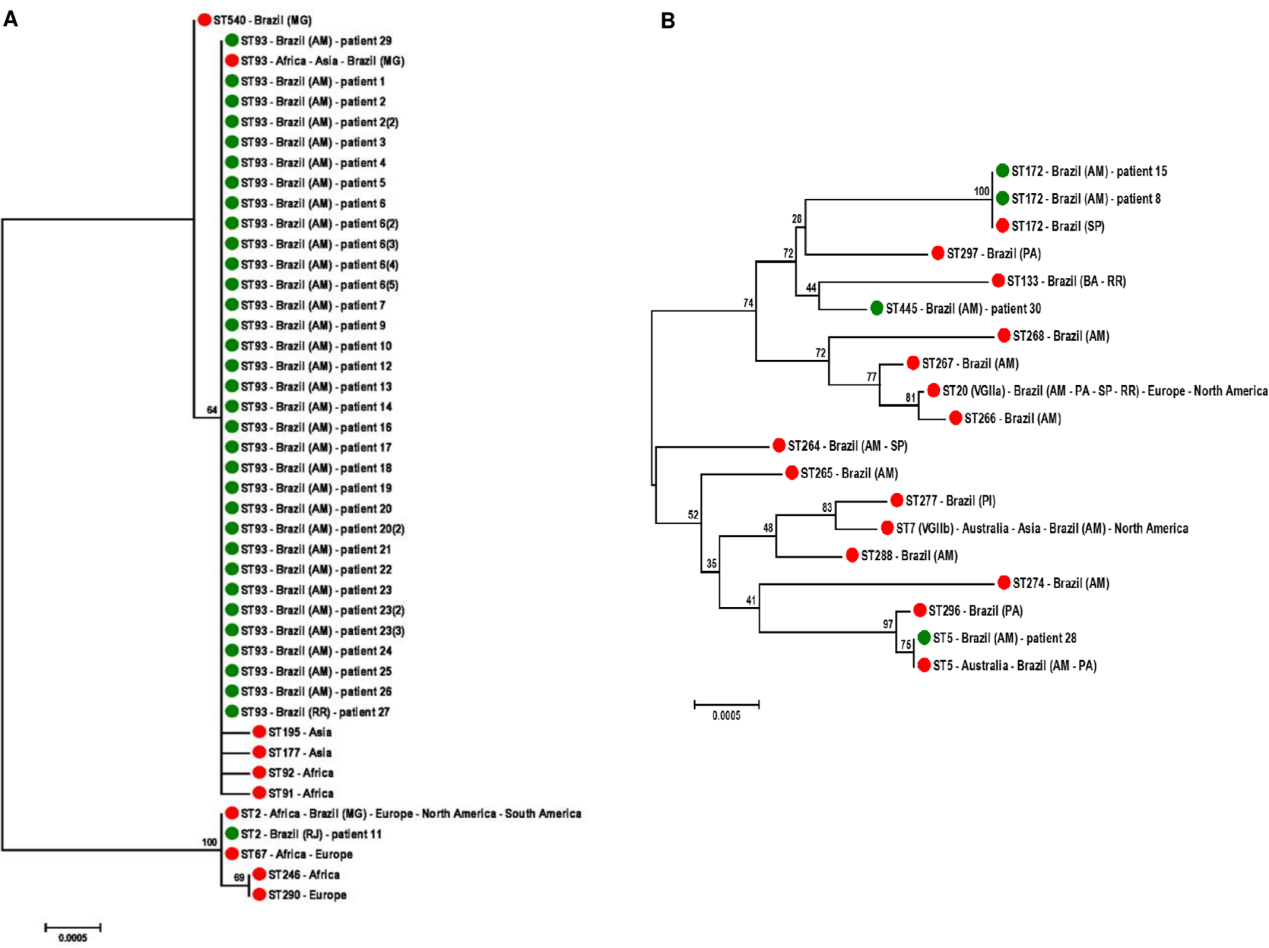
Unrooted neighbor-joining (NJ) trees constructed with the concatenated data set of seven MLST loci (*CAP59*, *GPD1*, IGS1, *LAC1*, *PLB1*, *SOD1* and *URA5*), showing the genetic relatedness of 34 VNI and 4 VGII STs of clinical isolates with those obtained from the Fungal MLST Database (http://mlst.mycologylab.org) and known geographic origin (only the closely genetically related STs were retained in the final tree). A) Phylogenetic analysis comparing the 34 VNI STs identified with 10 additional STS maintained in MLST Database. B) Tree representing the genetic association between three VGII STs defined in the present work with other Brazilian STs, including ST5, ST7, ST20, ST264, ST265, ST266, ST267, ST268, ST274 and ST288 found previously in Amazonas). The bootstrap values (1,000 replicates) are shown above the branches. Green circles and the patient code (S1 Table) were used to highlight the STs found in this study and red circles to indicate the STs retrieved from the MLST Database. A review of the literature was performed to check the geographical origin of VNI [31–37] and VGII [22,23,38,39] STs, described in the right side of the STs identification. The following abbreviations represent the Brazilian states: AM (Amazonas), BA (Bahia), MG (Minas Gerais), PA (Pará), PI (Piauí), RJ (Rio de Janeiro), RR (Roraima) and SP (São Paulo).

*URA5*-RFLP analysis identified 34 clinical *C*. *neoformans* as VNI and four *C*. *gattii* as VGII (S1 Table). In addition, MLST analysis divided these 38 strains into five STs. The *C*. *neoformans* VNI isolates were identified as the previously known ST93 (33, 97%), considered the most prevalent sub-genotype of opportunistic strains that affect immunosuppressed patients in northern Brazil. Only one strain presented as ST2, and this was recovered from the non-autochthonous case from Rio de Janeiro (Fig 1). Despite the few *C*. *gattii* VGII strains obtained, MLST identified three different STs as the previously known ST172 (2; 50%) and ST5 (1; 25%) and newly identified ST445 (1; 25%). No genotypic differences were observed among the strains recovered from serial isolates, as well as among the strains isolated from different clinical specimens, excluding the possibility of mixed infections (S1 Table). For the VNI isolates, the phylogenetic analysis demonstrated that ST91, ST92, ST177, and ST195 cluster tightly together with ST93 (Fig 2A), whereas ST133 of the VGII molecular type was the most closely genetically related to the new ST445 (Fig 2B).

### MIC results

The antifungal susceptibility test was performed with one strain per patient (n = 30). The antifungals AMB, FLZ and ITZ used to treat the patients showed satisfactory inhibition activity against all *C*. *neoformans* VNI and *C*. *gattii* VGII strains, which were considered wild-type (susceptible) isolates according to ECVs, although the VGII isolates showed a geometric mean (GM) four times higher than those of VNI. The highest MIC (32 μg/ml) for FLZ was observed in both ST172 strains of *C*. *gattii* VGII, although the number of samples per ST is very limited, precluding any conclusions. The MIC range and geometric mean of each drug tested are presented in Table 2.

**Table 2:** MIC ranges of the five STs identified and differences in the geometric means of the VNI and VGII strains.

Genotypes (total)	Amphotericin B					Fluconazole						Itraconazole					
	GM	MIC (μg/ml)				GM	MIC range (μg/ml)					GM	MIC range (μg/ml)				
		0.03	0.06	0.125	0.25		2	4	8	16	32		0.03	0.06	0.125	0.25	0.5
**VNI–ST93 (25)**	0.06	10	6	5	4	4.57	2	15	8	-	-	0.07	10	3	6	6	-
**VNI–ST2 (1)**		-	-	1	-		1	-	-	-	-		1	-	-	-	-
**VGII–ST5 (1)**	0.06	-	1	-	-	19.0	-	-	-	1	-	0.29	-	-	1	-	-
**VGII–ST172 (2)**		1	-	1	-		-	-	-	-	2		-	-	-	1	1
**VGII–ST445 (1)**		-	1	-	-		-	-	1	-	-		-	-	-	-	1

## Discussion

*C*. *neoformans* VNI and *C*. *gattii* VGII are the most common and clinically important agents of cryptococcosis in the state of Amazonas [24,40]. In the current study, some regional aspects of the molecular epidemiology of cryptococcosis were elucidated by applying the MLST. The results indicate that immunosuppressed patients from northern Brazil are affected by a clonal group of VNI strains represented by ST93 and ST2, which are globally isolated and genetically close to the African and Asian STs [32,35–37]. These are new findings for the Amazon region and demonstrate the low intragenotypic diversity of *C*. *neoformans* VNI. Although there were a limited number of *C*. *gattii* VGII strains analyzed, MLST revealed 3 subtypes in 4 strains, including a new subtype (ST445), indicating a higher genetic diversification of Brazilian VGII strains, as demonstrated previously [23]. We also report the antifungal susceptibility profile of STs from the Amazon region; however, it was not possible to establish correlations because of the small number of isolates analyzed.

Few studies on the epidemiology of cryptococcosis in Amazonas state (north of Brazil) have been performed, and the scarce data has shown the endemicity of cryptococcal meningitis caused by *C*. *neoformans* VNI in our region [3,24,40]. According to data obtained from records of the Mycology laboratory of the FMT-HVD in Manaus, cryptococcosis was the most frequent systemic mycosis diagnosed during the last 3 years, with an average annual occurrence of 33 cases, being more prevalent in HIV patients (82%). Comparing this information with previously published data from 2006 to 2008 [24], an increase of approximately 24% in the frequency of the disease was detected, but no significant differences in the epidemiological characteristics of the disease were observed. Meningoencephalitis caused by *C*. *neoformans* VNI remains the most important fungal infection in HIV male patients aged between 20–45 years (mean of 39 years old), as previously reported by regional surveys [3,24,40]. These same profiles were observed in the AIDS-associated cryptococcal meningoencephalitis cases reported in African and Asian cohorts, as well as in other Brazilian studies [1,4,32,41,42]. An epidemic of AIDS cases has occurred in the last few years in the Amazonas state and can explain the emergence of new cryptococcosis cases [4].

We also verified that 65% of HIV patients showed severe immunosuppression (CD4^+^ count <50 cells/mm^3^) at baseline, and all presented late diagnosis, with signs and symptoms of disseminated infection and neurological impairment, which may have contributed to the high lethality (54%) observed in the first hundred days after admission. The low adherence to antiretroviral therapy (HAART), and even the absence of early detection strategies for HIV and related opportunistic agents such as cryptococcosis, are factors that contribute to the occurrence of such injuries [4]. The use of an immunoassay for the detection of cryptococcal antigen (CrAg) incorporated into HIV testing would overcome this situation by screening asymptomatic patients with CD4 <200 cells/μl, allowing the initiation of pre-emptive treatment and preventing morbidity and mortality [43]. Its applicability in assessing the prevalence of antigenemia remains rarely studied in Brazil [44] and in Amazonas, where its use was recently initiated in the FMT-HVD for the investigation of some cases.

Despite the small number of patients evaluated, which is proportional to the annual occurrence of cryptococcosis in the region, the frequency of initial symptoms and the lethality that we describe is similar to larger studies conducted in areas with a high burden of cryptococcal meningitis, such as South Africa and other regions in Brazil. However, a lower proportion of deaths (28% after a year) were observed in HIV patients from the United States, which could be explained by early diagnosis or differences in the virulence of strains [35,41,45,46].

The altered mental status was described as a contributing factor for death in patients with AIDS-related cryptococcal meningitis, and in the current study, this condition was detected in 46% of cases, consistent with the lethality rate reported [41,45–47].

Visual impairment may occur as a secondary manifestation of cryptococcal meningitis in 20–40% of cases, both in patients with and without HIV, and a similar rate was also observed in the present study (36.6%) [41,46]. The causes are multifactorial and could be due to neuritis and compression of the optic nerve, direct parasitism, papilledema and increased intracranial pressure [48–50]. A low frequency of papilledema was observed, described in only 2 patients, one with and one without HIV. The use of a CSF shunt reported in 4 cases serves as an indication of complications due to an increase in intracranial pressure; however, the frequency of the visual deficit was greater, demonstrating that other etiological mechanisms could be involved, thus making it necessary to perform a more detailed investigation for a better understanding of its etiopathogeny.

We report that ST93 is the main sub-genotype of VNI strains that affect immunosuppressed patients in the north of Brazil. These results are consistent with data recently released by a unique Brazilian study that analyzed a greater number of isolates from Minas Gerais state and described a high prevalence of ST93 in individuals with AIDS, as well as in environmental samples [32]. This ST was also observed in AIDS cases from African and Asian countries, mainly South Africa and India, as highlighted by the phylogenetic tree (Fig 2A) [35–37]. Our findings provide indications that regional VNI strains show a low intragenotypic diversity. Similar results were described by two broad molecular investigations conducted in Asia, and this limited genetic diversity can be assigned to a lower ability to perform genetic recombination favoring the occurrence of clonal reproduction and expansion of these lineages [32,37,51]. However, VNI isolates from Africa are the most diversified compared with global isolates due to their ability to reproduce both clonally or sexually, which can lead to recombination and mutations in the genome and thus genotypic variability. Phylogenetic and population genetic analysis indicated that global VNI isolates descend from African strains and that pigeons facilitated their global dispersal, which explains the presence of ST93 on different continents, including the North of Brazil [32,35,51–53]. Whole genome sequencing also demonstrated that all *C*. *neoformans* lineages show multi-continental distribution, indicating the highly dispersive nature of this species complex [54].

Only 1 isolate of *C*. *neoformans* VNI presented as ST2, but it was not considered a local subtype, as it was isolated from a patient from Rio de Janeiro (Southeast region). This is the first clinical report of ST2 in Brazil because it was only identified in a single environmental sample in Minas Gerais, demonstrating that this subtype occurs in low frequency in this region [32]. The same ST was previously identified in Africa, Argentina, and the United States and shows a high prevalence in Germany (Fig 2A) [33–35].

Regarding the VGII isolates, we describe the identification of the new ST445 that presented a genetic relatedness with the exclusively Brazilian ST133 (Fig 2B), detected previously from a clinical source in Bahia state (northeast region) and from an environmental sample in Roraima, a neighbor state of Amazonas (north region) [23,39]. ST172 was identified in two cases of meningoencephalitis in patients from rural areas of Amazonas, one from Manaus and the other from the municipality of Jutaí in the southwest of the state. ST172 was previously identified in a clinical strain from São Paulo (southeastern Brazil) but was identified for the first time in the Amazon region [23]. ST5 was isolated from an HIV-negative male patient, who was a user of illicit drugs and died when treated with liposomal amphotericin. This ST was reported in Australia in a single veterinary isolate and was identified in Brazil only in the northern region in the states of Pará and Amazonas, where it is one of the most frequent subtypes, likely because it is better adapted to the local environmental conditions; however, there are no clinical data or evidence of virulence related to this subtype [23,38].

According to the ECVs, all isolates were considered sensitive to the three antifungal agents evaluated, although *C*. *gattii* VGII presented geometric mean values greater for the azoles than *C*. *neoformans* VNI, mainly to FLZ. ST172 demonstrated the highest MIC (32 μg/ml) for FLZ, but more strains must be analyzed before drawing any conclusion about the association between STs and drugs susceptibility. An association of the main genotype VGII with a lower susceptibility to FLZ has been widely described [55–57]. In Amazonas, a disc-diffusion assay noted the occurrence of two clinical isolates with FLZ resistance [24]. However, is important to screen out which VGII STs may be strongly associated with this reduced susceptibility, since the data are scarce and investigations with a greater number and diversity of STs are still necessary for an accurate correlation. Preliminary data were shown by Iqbal et al. (2010) [58], who demonstrated that distinct subtypes can present significant differences in MIC, as observed with the STs from the Pacific Northwest of the United States, with ST6 (VGIIc), ST7 (VGIIb) and ST20 (VGIIa) considered the least susceptible to azoles. Using a great number of multicentric isolates, Espinel-Ingroff et al. (2012) [29] presented additional and correlative data about the distribution of azoles MICs among VGII isolates, demonstrating that there may be variability in the intra- and inter-subtypes.

The higher geometric mean of FLZ for VGII isolates, as well as the variability in MIC values among identical STs, can be attributed to the mechanism of heteroresistance. Subpopulations of cells of a given isolate, independent of the pathogenic species, innately have the ability to duplicate chromosomes containing genes for FLZ resistance. Thus, they become able to tolerate increasing concentrations of this antifungal, developing a survival mechanism for such agents against the stress generated by the drug *in vitro* or *in vivo*, and evidence showed that heteroresistance in *C*. *gattii* is more pronounced than in *C*. *neoformans* [59–61].

The VNI isolates from this study were completely susceptible to the antifungal agents, and these data are consistent with the literature, including information obtained on VNI strains from Brazil and with previous data of Amazonas [6,24,55,62]. However, we demonstrated for the first time that ST93 strains from this state are sensitive to AMB and azoles, and these data are new for Brazil. Contradictory results were obtained by Khayhan et al (2013) [37] when analyzing the antifungal susceptibility of 52 Asian ST93 strains. They observed the occurrence of simultaneous resistance to FLZ and to flucytosine in 5 isolates from Indonesia. In comparison, it is possible that similar STs may have different susceptibility profiles, likely due to the limitation of the MLST methodology, which analyzes 7 loci in the genome. Moreover, the environmental and climatic differences in different regions may influence the antifungal response [57].

## Conclusion

In conclusion, *C*. *neoformans* VNI strains from Amazonas are a genetically monotypic group that commonly presented as ST93, but further whole genome analysis should be performed to confirm this genetic homogeneity. The ST93 showed great clinical and epidemiological importance due to the frequent morbidity and lethality associated with cryptococcal meningoencephalitis in individuals with AIDS in this state and likely in northern Brazil. The establishment and predominance of ST93 in Amazonas may have been favored by isolated events of genetic recombination in its African ancestors, which later spread to several continents and maintained a clonal expansion mechanism. The three VGII STs identified showed genetic association with Brazilian STs; however, they did not cluster with others STs from Amazonas. Based on the MIC values obtained under *in vitro* conditions, all isolates were considered susceptible to the antifungal drugs evaluated, but the use of FLZ deserves attention due to its limited ability to inhibit the growth of VGII strains, and these data can be predictive of clinical failure.

## Supporting information

S1 Table Patient information and genotyping data of all isolates analyzed. (XLSX)
